# Fetal syringomyelia

**DOI:** 10.1186/s40478-014-0091-0

**Published:** 2014-08-06

**Authors:** Anne Guo, David Chitayat, Susan Blaser, Sarah Keating, Patrick Shannon

**Affiliations:** Department of Pathology and Laboratory Medicine, 6th Floor, Mount Sinai Hospital, 600 University Avenue, Toronto, Ontario M5G 1X5 Canada; Department of Clinical Genetics, Mount Sinai Hospital, Toronto, Canada; Department of Diagnostic Imaging, Hospital for Sick Children, 555 University Avenue, Toronto, Ontario M5G 1X8 Canada

**Keywords:** Syringomyelia, Fetal, Chiari II malformation, OEIS, Dysraphism, Spinal cord

## Abstract

We explored the prevalence of syringomyelia in a series of 113 cases of fetal dysraphism and hindbrain crowding, of gestational age ranging from 17.5 to 34 weeks with the vast majority less than 26 weeks gestational age. We found syringomyelia in 13 cases of Chiari II malformations, 5 cases of Omphalocele/Exostrophy/Imperforate anus/Spinal abnormality (OEIS), 2 cases of Meckel Gruber syndrome and in a single pair of pyopagus conjoined twins. Secondary injury was not uncommon, with vernicomyelia in Chiari malformations, infarct like histology, or old hemorrhage in 8 cases of syringomyelia. Vernicomyelia did not occur in the absence of syrinx formation. The syringes extended from the sites of dysraphism, in ascending or descending patterns. The syringes were usually in a major proportion anatomically distinct from a dilated or denuded central canal and tended to be dorsal and paramedian or median. We suggest that fetal syringomyelia in Chiari II malformation and other dysraphic states is often established prior to midgestation, has contributions from the primary malformation as well as from secondary in utero injury and is anatomically and pathophysiologically distinct from post natal syringomyelia secondary to hindbrain crowding.

## Introduction

The association between syringomyelia and malformative conditions, and in particular the Chiari malformations has been examined in some depth, with a wealth of clinical, radiological, pathological, surgical and experimental studies see, for review [1-6]. Current ideas about syrinx formation centre on alterations in cerebrospinal fluid flow near the craniocervical junction and alterations in vascular and interstitial fluid dynamics, which by a variety of proposed mechanisms are thought to result in an accumulation of fluid within the cord, and subsequent intramedullary cavitation or dilation of the central canal [1,3,7]. In these conceptions, with a few exceptions, [6], syringomyelia is often thought to be a chronic, progressive, and late consequence of the underlying malformation or acquired disease [1,5,8,9]. Syringomyelia in the context of terminal cord myelocystocele, on the other hand, is proposed to be a straightforward ballooning of the terminal ventricle in communication with a patent central canal [10-12].

Despite the wealth of early pathological descriptions in the post-natal and paediatric literature e.g. [4,5,13-15], there is only a scant literature on the pathology or mechanisms of syrinx formation in the human fetus. This is perhaps surprising, as large post-mortem series on the subject mention syrinx formation as not infrequent in fetal, neonatal and paediatric Chiari II malformations [14,15] and there is an ultrasound report of detection of syringomyelia in a pre-term fetus [16], although a large radiological series demonstrates detection by MRI be very unusual and late in gestation [17].

In general, a syrinx can be characterised pathologically as an abnormal, fluid filled within the substance of the spinal cord that may or may not be lined by ependyma, and distinguished from the normal central canal by its location, surrounding reactive glia and disruption of adjacent structures [2]. Syringes have been classified into two major morphologies: the communicating, or hydromyelic form, and the paracentral, or syringomyelic or non-communicating form [3-5,18]. The hydromyelic form is generally thought of as a distention of a patent central canal in communication with the IVth ventricle, and is often associated with the Chiari II malformation. Such syringes are generally lined at least in part by ependyma, but may be irregularly denuded. The syringomyelic form is more common in Chiari I malformations, as well as in other lesions obstructing cerebrospinal fluid flow at the craniocervical junction without dysraphism, and is generally represented by an irregular transverse cleft, sometime intersecting the central canal but not representing a mere distension of the canal. In both forms, the abnormal fluid dynamics near the cranio-cervical junction are thought to lead to an increase in trans-pial pressure gradients and an increase in extracellular fluid [1,3,7].

In our practice of fetal neuropathology, we have occasionally encountered forms of spinal cord cavitation accompanying dysraphism which do not neatly fit into these categories and distinction between hydromyelic and syringomyelic pathologies may be difficult or arbitrary. Given the importance of the association between malformation and syringomyelia, it would seem obvious that ideas about syrinx formation in developmental abnormalities might be clarified or advanced by studying syringomyelia as early as possible in its genesis, and therefore human fetal material might be of some value. We therefore systematically reviewed our experience with fetal syringomyelia over ten years in an autopsy service specialising in fetal anomalies, high risk pregnancy and genetic disease in order to clarify the anatomy and clinical correlates of fetal syringomyelia.

## Materials and methods

We reviewed our experience over 10 consecutive years, using the autopsy records for all cases of syringomyelia and hydromyelia. The histological diagnosis of syringomyelia is not wholly straightforward, as mild dilations of the central canal can occasionally be seen in the absence of any other central nervous system pathology. Indeed, for some radiologists the preferred term for extensive, centrally placed fluid collections in the setting of Chiari II malformations is hydrosyringomyelia [2]. However, many definitions of syringomyelia denote that the abnormal fluid filled space is accompanied by a distortion or injury of adjacent structures [2,18]. Accordingly, we define longitudinal cavitations as syringes if they are within the substance of the spinal cord, with an adjacent reactive glial component, if they are distorting the normal anatomy of the cord and are at least partially outside of the normal central, symmetrical location of the central canal.

When syringomyelia was noted in the diagnostic report, the histology and case records were retrieved. If there was any associated central nervous system malformative sequence or syndrome, the archives were also searched and all other cases with that associated condition were retrieved and the histology reviewed. This resulted in the examination of all Chiari type II malformations, Meckel Gruber syndrome, myeloceles, meningoceles, myelomeningoceles and the omphalocele/exostrophy/imperforate anus/spinal abnormality (OEIS) complex. Given the known associations of syringomyelia, all cases of spinal or cerebral dysraphism or congenital hindbrain crowding such as iniencephaly, encephalocele, Dandy Walker malformation, posterior fossa cyst, dural sinus malformation, and congenital cerebellar tumors were also retrieved and examined histologically. At our institution our standard procedure is to extract the entire spinal cord in all autopsy cases where consent is provided. In cases less than 24 weeks gestational age, the spinal column is removed with the cord in situ. Where there is known or suspected spinal pathology, the entire cord is submitted at 3 mm intervals. Otherwise, at least 5 sections of spinal cord are examined. Sections are fixed embedded and stained according to routine protocols, and where appropriate, immunohistochemistry for epithelial membrane antigen, glial fibrillary acid protein (GFAP), alpha beta crystallin, neurofilament light chain, myelin basic protein, nestin, vimentin, CD168 and CD63 are employed. We excluded cases where autolysis or mechanical artefact made interpretation ambiguous or difficult, or if sampling of spinal cord and column did not follow standard procedure.

## Results

The study period encompassed 1,965 neuropathological examinations, amoung which we encountered 112 cases of dysraphism. From these we retrieved 51 cases of Chiari 2 malformation, of which 5 were not included due to inadequate sampling (one case) or excess autolytic and mechanical artefact (4 cases). All the remainder demonstrated open neural tube defects in the form of lumbosacral or thoracolumbar myelocele in addition to the usual posterior fossa abnormalities. One displayed in addition an occipital encephalocele. The gestational age ranged from 17.5-24 weeks, and all specimens were the result of pregnancy termination following the detection of fetal anomalies by ultrasound. Forty two of the remaining 46 demonstrated myeloceles with myeloschisis with two demonstrating occipital encephalocele, one lumbosacral myelomeningocele and one thoracolumbar myelocystocele. All demonstrated mild enlargement of the central canal outside of the area of spina bifida. Among these cases we identified 12 syringes, all of them in the context of myelocele and myeloschisis. Syringes followed three major morphological distributions as follows.

**Figure 1:**
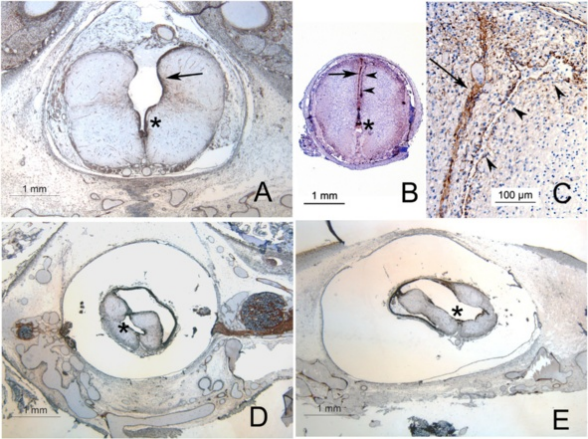
**Syrinx morphology in Chiari II malformations. A** Type 1 syrinx: the ventral portion of the cavity is ependymal lined (asterix), while the dorsal aspect is irregularly lined by a layer of hyperplastic glia (arrow). Despite the lack of dorsal ependymal lining, the impression that this is simply a wedge shaped extension of the more caudal dysraphism is difficult to escape. **B** Type 2 syrinx, extending in this section into the cervical cord, with a prominent paramedian and dorsal distribution. The arrow indicates the normal dorsal and median glial raphe and arrowheads the slit-like paramedian syrinx. This cavity was continuous with the dilated central canal (asterix) caudally. **C** Dorsal aspect of the same section as **B** at higher power, demonstrating glial processes lining a slit like cavity containing macrophages, with axonal tracts in the walls of the cavity. **D** and **E**: Same specimen, type 3 syrinx, demonstrating irregular extension of the syrinx both dorsolaterally **(D)** and ventrolaterally **(E)**. The cavity marked by an asterix is partially lined by ependyma and is continuous with the central canal. All sections stained immunohistochemically for vimentin.

In 4 cases of Chiari II malformation, the cavity which we will term a Type 1 syrinx was a cavity dorsal to the central canal, with its apex at central canal and its base at the dorsal pial surface of the cord (Figure 1), extending rostrally 2–3 levels from the site of spina bifida and in communication with the central canal. In one such case there was a very large myelocystocele, with the dorsal elements of the cord severely attenuated and extending as a subcutaneous cyst in continuity with underlying spina bifida, extending from the rostral sacrum to the lower cervical spine. The dorsal elements of this cyst were composed of an ependymal lined membrane adherent to arachnoid applied directly to the subcutaneous connective tissue, and the ventral aspect of the was the splayed open, flattened spinal cord.

In 8 cases, there was a syrinx morphology we refer to as a type 2 syrinx in which the syrinx varied in shape from wedge shaped to slit like, was in continuity with the central canal only for one or two levels adjacent to the myelocele, and with one exception extended rostrally from the myelocele, and generally had a paramedian to midline course, either within or between the dorsal columns (Figure 1). In four of the 8 cases of type 2 syrinx, the syrinx ran the length of the cord and also connected to the central canal at the cervicomedullary junction. In three more it was thoracolumbar arising from a lumbosacral myelocele, and in the last it extended from a thoracolumbar myelocele rostrally into the cervical cord. In these latter 4, the syrinx did not therefore connect again with the rostral central canal or the IVth ventricle.

A third form of syrinx (type 3) was present in a single case and was a large irregular cavity extending nearly the length of the cord into the lower cervical levels, dissecting irregularly and laterally into the cord substance, accompanied by severe atrophy of the neural structures and continuous at multiple points along its length with a dilated and distorted central canal (Figure 1). None of the cases demonstrated a simple multisegment cystic dilation of the central canal. In all cases, the dorsal aspect of the syrinx cavity is represented by a thin membrane containing glial and neural elements with overlying arachnoid.

**Figure 2:**
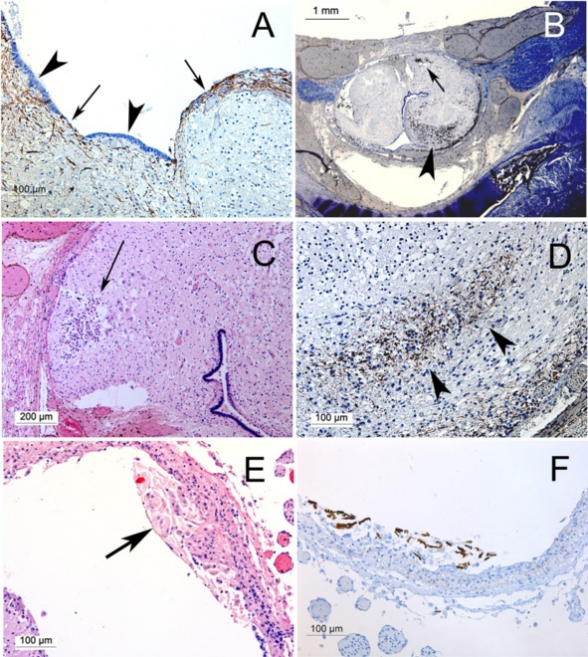
**Spinal cord pathology associated with syringes in Chiari II malformations. A** The anatomical boundary between syrinx and central canal may be indistinct, with syringes partially lined by ependyma (arrowheads) alternating with areas of glial hyperplasia (arrows, Immunohistochemistry for nestin). **B** Secondary changes in a spinal cord with syrinx: macrophages forming dense aggregates (arrow) and accumulating within the neuropil (arrowhead, immunohistochemistry for CD168). **C** H + E of same case as **B**, demonstrating aggregate of foamy macrophages. **D** Region of gliosis at area indicated by arrowhead in **B** (immunohistochemistry for alpha beta crystallin). **E** Vernicomyelia with foreign body macrophages (arrow). **F** Immunohistochemical reactivity of vernix to low molecular weight keratin (Cam 5.2).

The morphology of the syrinx lining was variable: particularly the dorsal aspect was often discontinuously lined by ependymal glia with round nuclei and a cuboidal profile, as opposed to the distinctly columnar ependyma of the central canal. Elsewhere, the cavities were lined by astrocytic glia or their processes, expressing GFAP, Vimentin, Nestin and more inconstantly alpha beta crystallin (Figure 2).

Among the cases of syringomyelia in Chiari II malformation, 7 of the 13 displayed vernicomyelia, i.e. the presence of amnionic squames in the syrinx cavity or in the spinal subarachnoid space with an associated histiocytic reaction (Figure 2). One was a type 1, five were type 2, and one was a type 3 syrinx. No case of Chiari malformation demonstrated vernicomyelia without syringomyelia. In the single case of a type 3 syrinx, vernix and reaction was present in the IV^th^ and lateral ventricles as well. In one case of type 2 syrinx the cord showed what we interpret as infarct like pathology and scarring, with an aggregate of foamy macrophages in one area and with patches of gliosis with microglial aggregation in others.

**Figure 3:**
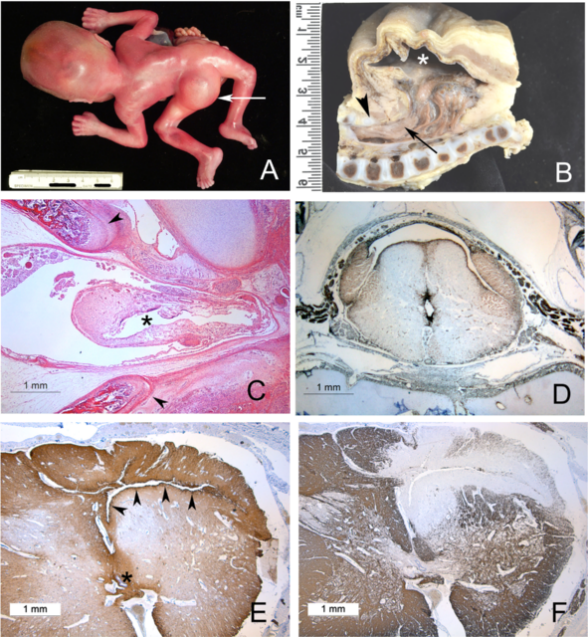
**Spinal cord and syrinx pathology in OEIS. A** OEIS in a midgestation fetus with large dorsal myelocystocele (arrowhead). **B** Longitudinal section of caudal spine and myelocystocele in a near term infant with OEIS. The dorsal sac (asterix) is lined by ependyma and continuous with the central canal of the spinal cord (arrow) which herniates dorsally. The dorsal elements of the neural canal are absent in the midline below the upper lumbar spine (arrowhead) and the conus is low lying. **C** Same specimen as **(A)**, whole mounted axial section of spinal column at the point where the cord herniates through dorsally between the separated spinal laminae (arrowheads). The central canal (asterix) is dilated and irregular with a discontinuous lining. **D** Axial section of cervical spinal cord of same specimen as **(A)** demonstrating dorsal syrinx with lateral extensions separate from a mildly dilated central canal (immunohistochemistry for vimentin). **E** Transverse section through the cervical cord of the same fetus as **(B)**, demonstrating an irregular dorsolateral cavity with adjacent increased staining for alpha beta crystallin (arrowheads), separate from normal sized central canal (asterix). **F** Same specimen and level as **(E)**, stained for myelin basic protein, with near total loss of myelin staining adjacent to the syrinx cavity.

We encountered 3 cases of isolated frontal encephalocele, none with syringomyelia. Among 25 cases of occipital encephalocele without Chiari malformation or spinal dysraphism (Median age 22 weeks, range = 18 to 26 weeks), two had syringomyelia, and both were in the context of Meckel Gruber syndrome. Of these two cases, both involved the cervical cord: one was isolated to the cervical cord, and corresponded to a type 1 syrinx, and one was a type 2 syrinx, extending the length of the cord (Figure 3) with both paramedian and dorsolateral cavitations, none of which were ependymal lined. At the margin of this syrinx were scattered pigmented histiocytes which stained positively for iron (Figure 3).

**Figure 4:**
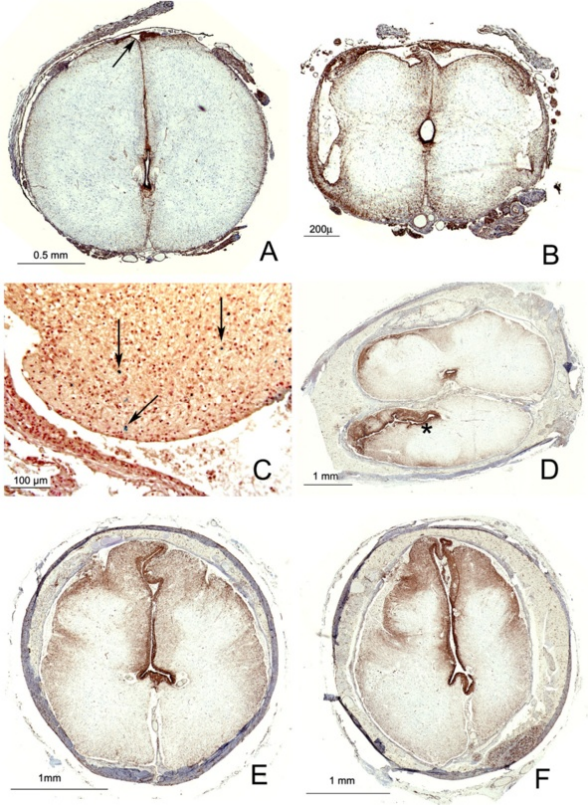
**Syringes in Meckel Gruber and pyopagus twins. A** Cross section of cervical spinal cord, Meckel Gruber with an occipital encephalocele. **A** small dorsal median cavity (arrow) is continuous ventrally with the dorsal glial raphe, but does not appear to connect to the mildly dilated and dorsally elongated central canal in this plane. **B** Same case, thoracolumbar cord: large dorsal syrinx with irregular lateral extensions (**A** and **B**: Immunohistochemistry for Vimentin). **C** Wall of syrinx in **(B)**, with scattered histiocytes reacting positively for Iron (arrows, Perl’s iron stain). **D** Sacral spinal cords of pyopagus twins stained for alpha beta crystallin: one cord is a hemicord with a collapsed and dilated central canal (asterix). Both cords share a thin fibrolipomatous sheath within a single dural sac. More rostrally, the lumbar spinal cords of each twin separated and had distinct dural sacs within a common neural canal. **E** Cross section of thoracolumbar spinal of the cord oriented as the superior cord in **D** demonstrating a dorsally elongated central canal lined by ependyma and extending between the posterior columns. **F** The abnormality in **(E)** is duplicated in the thoracolumbar cord of the other twin.

We retrieved eight cases of the omphalocele exostrophy imperforate anus sacral anomaly syndrome (median age = 21 weeks, range = 18 to 34 weeks). None displayed a concomitant Chiari malformation. All had sacral dysraphism with low lying conus and a dilated sacral central canal. Five of the eight displayed a large dorsal myelocystocele with ascending syringomyelia (Figure 4). The myelocystocele is composed of a dorsally expanding cyst wherein the dorsal aspect of the cyst is composed of ependyma resting on arachnoid adherent to subcutaneous fibrous tissue, and the ventral aspect is an opened, flattened cord. In two cases the syrinx extended rostrally to the mid-cervical spinal cord. The ascending syringomyelia is in no case ependymal lined, though caudally it is in continuity with the myelocystocele. In one case, where the lumbosacral spinal cord herniated dorsally though the absent neural arch, there was near complete local cavitation of the spinal cord, with reactive gliosis in the remaining cord, and a pattern of syringomyelia similar to that seen in one of the cases of Meckel Gruber, with paramedian and dorsolateral cavitation. The other cases of hindbrain crowding or neural tube defect included 23 cases of anencephaly and 12 of the Dandy Walker malformation, five dural sinus malformations and two congenital cerebellar tumors, and a single case of lumbosacral spina bifida occulta with a very low lying conus without a Chiari malformation, none of which had syringomyelia.

One instance of sacral dysraphism was distinct: this case comprised the abnormal spinal cords conjoined twins (Figure 4) fused dorsally, with their lumbosacral spinal cords sharing a common neural canal. The lumbosacral segments of both cords shared a sheath of fibrolipomatous tissue, but were otherwise separate along their lengths. Both cords had dilated central canals which in the thoracic and cervical sections assumed a dorsal, slit like profile extending between the dorsal columns. In those sections the dorsal aspect was only devoid of ependyma in small patches but remained in continuity ventrally with the expanded central canal.

## Discussion

This series demonstrates that syringomyelia is not uncommon in fetal autopsy material, if sought carefully, and can be occasionally expected as part of the suite of several malformative conditions by midgestation. The presence of a spinal cord syrinx in Chiari II malformations in neonatal and fetal specimens has been documented [4,5,13-15] but it is usually not clear how or whether the authors distinguish between syringomyelia and a persistently patent central canal, as the histomorphological distinction between the two is not explicitly defined. Moreover, the morphology and extent of the syringes reported is generally poorly documented. In our series, the fetal syringes are continuous with the site of the dysraphism, are in continuity with the central canal for at least part of their length and can therefore be classified as communicating syringes as one might expect.

In a minority subset of Chiari II malformations, (i.e. the Type 1 syringes, and in the cavities within the pyopagus twins (Figures 1 and 4) the impression is that the cavities represent a local hypoplasia of dorsal elements and a concomitant expansion of the central canal. The Type 1 syringes would thus represent the rostral end of the area of incomplete dorsal fusion of the cord. However, in the remainder of the syringes, these cavities are not simply expanded central canals, as their morphology is radically different from the normal central canal, they may have prominent non-central components, and there is an obvious disturbance of the normal neural structures. Such syringes were not continuous with the central canal along their entire extent, all were associated with surrounding gliosis, and some were associated with definite evidence of secondary antemortem destructive lesions, such as inflammation (i.e. vernicomyelia) or infarct, apparently acquired in utero. These syringes sometimes had multiple areas that were separated from the central canal by white matter tracts but were still partially lined by ependyma. The consistently dorsal median and paramedian aspect of these syringes, their thin dorsal walls and their partial ependymal linings suggest that they are remnants of the central lumen of a neural tube with dorsally hypoplastic elements, as one might expect in a dysraphic spinal cord. However, the presence of axonal tracts separating these cavities from the central canal suggests that the establishment of axonal tracts in the wall of the neural tube may contribute to the establishment of eccentric cavities that represent a partially filled in, abnormal neural tube canal.

In this series, syringomyelia is present in a number of dysraphic conditions. Moreover, the syringomyelia in each case was predominantly associated with the region of the herniation of the neuraxis through dorsal bony structures: in the Chiari II malformation and OEIS, congenital syringes ascended from the site of spina bifida, while in both cases of Meckel Gruber, the syringes were in part cervical, and adjacent to the occipital encephalocele. This geographical continuity with dysraphism, together with the evidence of infarction and old haemorrhage in some of these cords, suggests that herniation of the spinal cord may lead to local tissue injury and predispose to cavitation. One published series of fetal myeloceles suggests that injury secondary to delivery is the dominant spinal cord lesion in the presence of an open neural tube defect [19]. Indeed, agonal haemorrhage without tissue gliosis or reaction is very common in all early fetal neuropathological specimens. However, such acute haemorrhage will not produce the gliosis surrounding these cavities that we illustrate here, and the cavities illustrated do not contain blood, and we conclude that these lesions are not secondary to parturition, but are a consequence of the malformative sequence as well as early secondary events. Although the fetal cord is a fragile pathological specimen, and can be distorted by autolysis and artefact, our examination technique of whole cord spinal column removal and decalcification with the spinal cord *in situ* minimises distortion, and because we required the presence of a reactive glial lining in order to identify a syrinx, we discount the possibility that the cavities we observe are artefactual.

The prevalence of vernicomyelia among the cases of Chiari malformations with myelocele deserves comment. Vernicomyelia can be a spectacular and striking feature in fetal specimens [20-23]. Possibly the high prevalence in this series reflects the early gestational age: the available autopsy series exploring the subject demonstrates an increased prevalence of vernicomyelia among fetal and neonatal post mortems as compared to young children [20]. The reason for this age difference is unclear. Perhaps the sampling of the complete neuraxis is rendered easier by the sheer size of the younger fetus, or the offending vernix is cleared during post natal development, or that the most severe pathologies are over-represented in pregnancy termination and neonatal deaths. It may be that vernicomyelia is only possible in the presence of a significant syrinx that allows reflux of the vernix into the open defect. The high prevalence of vernicomyelia in the presence of syringomyelia, and the absence of vernicomyelia in the absence of syringomyelia suggest that either the secondary inflammatory pathology of vernicomyelia plays a role in generation of spinal cord pathology, or that vernicomyelia is a marker for severe pathology. Either way, by mid gestation vernicomyelia is prevalent and associated with severe injury in this series. This suggests that injury to the cord by vernicomyelia is unlikely to be wholly prevented by midgestation fetal surgical repair, though indeed such surgery might prevent further injury.

Among the central nervous system malformations that accompany syringomyelia are chronic hindbrain crowding conditions, such as the Chiari and Dandy Walker malformations [5]. In these conditions, a wealth of radiological, surgical, clinical and experimental work points to the conclusion that syrinx formation is due to altered cerebrospinal fluid dynamics near the craniocervical junction, and suggests that syringomyelia is a late, slowly progressive phenomenon [1,5,8,9]. Typical hydromyelic syringomyelia, as documented primarily in infants and children, may develop by term gestation as a consequence of chronically altered cerebrospinal fluid dynamics, leading to a progressive expansion of the central canal in utero. However, such an explanation does not completely account for the morphology we see in this series. The syringes present in this series are established by midgestation and do not consist of a dilation of the central canal: many appear to have a prominent developmental component with a failure of complete neural tube closure, hypoplasia of dorsal structures, and the irregular establishment of axonal tracts in the walls of the syrinx (Figures 2 and 3) separating the lumen of the syrinx from the central canal. Moreover, the presence of inflammatory pathology and features suggesting ischemia or haemorrhage suggests that the abnormal anatomy renders these cords vulnerable to secondary insults prior to labor and delivery. Syringomyelia in dysraphic states may therefore have a substantial developmental component and is at least morphologically distinct from that presenting in later life.

We are not proposing that syringomyelia in dysraphic states in general is due to purely local abnormalities in cord development. Syringomyelia does not appear to be associated with anencephaly in this series, so dysraphism per se is not a sufficient explanation for the syringomyelia. However, the presence of secondary pathology superimposed on a dysraphism suggests that the pathogenesis of syringomyelia, at least in the context of significant malformation, requires re-evaluation and the contribution of in utero insults be considered. We note that the presence of significant syringes with variable morphology in the human fetus in the presence of an open neural tube defect makes the current usage [6] of “embryonic” syringomyelia (representing a dorsal dilation of the neural tube) and “fetal” syringomyelia (associated with intact dorsal bony elements) a confusing nomenclature as the syringes we encounter in the human fetus have both a malformative and a secondary component, and may be associated with either open or closed dysraphism.

Our results stand in contrast to a recent MRI study [17] that demonstrated syringomyelia in only 1.1% of fetal open neural tube defects, and then only late in gestation, and so suggested that the term congenital syringomyelia may be obsolete. However, the Chiari II malformations in our series are exclusively in mid gestation fetuses, and roughly one-fifth demonstrate syringomyelia. It should be noted that the majority of syringes in the open neural tube defects in our series are slit like, less than 1 mm in diameter, and in spinal cords generally less than 4 mm in diameter (e.g. see Figure 1B and C). We suggest that part of the discrepancy between our results and the radiological series is simply the higher resolution of histology compared to MRI. It may be that the usual hydromyelic form of syringomyelia documented primarily in infants and children results from a progressive expansion of the central canal, and is not generally seen until term or early post natal life, and fetal syringes may expand in post natal life. Also, this series is drawn largely from pregnancy terminations and the overwhelming predominance of myelocele with myeloschisis suggests that intrinsic developmental abnormalities of the spinal cord are probably more severe in this series than in the population of Chiari II malformations in general.

The morphology of the spinal cord abnormality in OEIS is sparsely described in the pathology literature [11,12,24]. To our knowledge, ours is the only study to recognize and demonstrate an anatomical distinction between the caudal hydromyelia of OEIS which is common, and the dramatic ascending syringomyelia distinct from the central canal illustrated here. However, in none of these cases was there an obstruction to cerebrospinal fluid circulation either in the spinal canal or at the craniocervical junction except possibly caudally, at the point of dorsal herniation of the cord, nor was there a reason to suspect reduced craniospinal compliance [7]. However, the syringes ascend in the cord, and do not communicate with the cervical central canal or reach the level of the medulla. Common to all five cases of syringomyelia in this series is the presence of a dramatic dorsally dilated myelocystocele with hydromyelia, and an ascending syrinx in continuity with the cystocele caudally but rostrally diverging from the central canal. The pattern of ascending syrinx from a thin walled terminal bulb suggests as a speculative possibility that in terminal myelocystoceles a syrinx might be formed by fluid forced into the substance of the spinal cord by compression of the bulging dorsal sac, somewhat in the manner of fluid being forced from a turkey baster or bulb pipette.

In summary, syringomyelia in fetal specimens is an unusual, but not remarkably infrequent accompaniment to a variety of dysraphic states. The pathogenesis of syringomyelia in Chiari II malformations and other congenital conditions deserves some re-appraisal, as the morphology is not consistent with the effects of simple alterations of fluid dynamics, nor is syringomyelia a late complication here. Our series suggests that syringomyelia can be acquired in the first half of gestation, and represents a combination of intrinsically abnormal development, secondary pathology and possibly superimposed and contributing abnormal fluid dynamics. The contribution of in utero insults to the development of clinical syringomyelia in the post natal state is not a topic well addressed and will require careful evaluation if fetal surgical therapy for dysraphism is to become common.
